# Lower normal free thyroxine is associated with a higher risk of metabolic syndrome: a retrospective cohort on Chinese population

**DOI:** 10.1186/s12902-021-00703-y

**Published:** 2021-03-04

**Authors:** Xi Ding, Chun-Ying Zhu, Rui Li, Li-Ping Wu, Yue Wang, Shi-Qian Hu, Yi-Ming Liu, Feng-Yi Zhao, Yang Zhao, Meng Zhang, Ming-Qian He, Zi-Yi Chen, Bing-Yin Shi

**Affiliations:** 1 Department of Endocrinology, First Affiliated Hospital of Xi’an Jiaotong University, 277 Yanta West Street, Xi’an, 710061 China; 2 Department of Disease Prevention And Control, Shaanxi Xi’an Electric Power Center Hospital, Xi’an, China; 3 School of Public Health, Xi’an Jiaotong University, Xi’an, China

**Keywords:** Lower normal free thyroxine, Thyroid stimulating hormone, Metabolic syndrome, Retrospective cohort study

## Abstract

**Background:**

Recently, the relationship between thyroid hormones (THs) across the euthyroid ranges and metabolic syndrome (MetS) has been widely discussed. This study aimed to present specific cutoff values of THs to assess the association between THs and MetS in a euthyroid cohort.

**Methods:**

Data of 2694 subjects, aged 18–80 years, who attended health examination in Xi’an Electric Power Central Hospital from April 2011 to December 2015 were collected and analyzed. The first cohort enrolled 929 participants (followed up by 2221 person-years totally) to assess correlations between serum thyrotropin (TSH), triiodothyronine (T3), thyroxine (T4) levels and MetS. The second cohort included 698 participants (followed up by 1709 person-years totally) to evaluate relationships between serum free triiodothyronine (FT3), free thyroxine (FT4) levels and MetS. MetS was defined according to the criteria of the American Heart Association/National Heart, Lung, and Blood Institute (AHA/NHLBI) scientific statements of 2009. Euthyroidism was defined as serum TSH, FT3 and FT4 levels within the reference ranges without taking any thyroid medication.

**Results:**

The cutoff values for TSH, T3, T4, FT3 and FT4 were 2.0mIU/L, 1.9 nmol/L, 117 nmol/L, 4.3 pmol/L and 16 pmol/L, respectively. Participants were categorized into two groups according to cutoff values: the lower-THs group and the higher-THs group. There was no significant difference in the risk of MetS between two groups in TSH, T3, T4 and FT3. The incidence of MetS was significantly higher in lower-FT4 group than higher-FT4 group (1.00 vs 0.622 (0.458, 0.846), *P* = 0.002). The lower-FT4/higher-TSH group had the highest hazard ratios of MetS. (2.131vs 1.0 (1.380,3.291), *P* = 0.006).

**Conclusions:**

Lower normal FT4 (FT4 ≤ 16.0 pmol/L) is an independent risk factor for MetS, and lower normal thyroid function (TSH > 2.0 mIU/L and FT4 ≤ 16.0 pmol/L) is associated with a higher risk of developing MetS.

**Supplementary Information:**

The online version contains supplementary material available at 10.1186/s12902-021-00703-y.

## Background

Metabolic syndrome (MetS) is a complex disease clustering of risk factors for cardiovascular diseases (CVD) that includes dysglycemia, high blood pressure (BP), high triglyceride (TG) levels, low high-density lipoprotein cholesterol (HDL-C) levels, and obesity (particularly central adiposity) [1]. MetS is associated with twice the risk of CVD and a 5-fold increase in risk of developing type 2 diabetes mellitus [1]. The prevalence of adults MetS is estimated to be relatively high worldwide, 35% in the United States [2] and 33.9% in China [3]. With adverse consequences and notable prevalence, MetS has emerged as a global health issue.

Thyroid hormones (THs) have been widely reported to be associated with MetS as they have multiple effects on energy homeostasis and metabolism [4]. THs signaling modulate energy expenditure through both central and peripheral mechanisms at different levels [5]. THs regulate metabolic rate and body weight through stimulating the expression of uncoupling proteins in the mitochondria of adipose tissue and skeletal muscle and enhancing the responsiveness of catecholamines to modulate adrenergic receptor numbers [6]. Moreover, THs have been confirmed to be correlated with insulin resistance [5, 7–10], body mass index (BMI) [10], lipid and glucose metabolism [7, 10], and BP [11], which are indispensable features comprising MetS.

Studies have previously investigated the relationships between subclinical hypothyroidism (SCH) and the prevalence or incidence of MetS. Gradually, it has been found that the fundamental pathophysiological mechanisms behind the effect of thyroid dysfunction on metabolic parameters may extend into the euthyroid status. More and more researches have focused on the correlations between THs across the normal reference ranges and MetS, especially suggesting an association between lower normal thyroid function and MetS.

Lower normal thyroid function is referred to as a higher normal TSH and a normal FT4, or a normal TSH and a lower normal FT4, or a higher normal TSH and a lower normal FT4. Lower normal thyroid function is associated with increased cardiometabolic risk factors and MetS due to increased insulin resistance [12].

Studies have demonstrated that high normal TSH is a risk factor for MetS in a euthyroid young Korean women population [13] and in a Chinese population over 40 years old [14]. In addition, one study found that the prevalence of MetS significantly decreased from 30.1% in the lowest FT4 tertile to 22.4% in the highest FT4 tertile in euthyroid subjects, indicating that lower normal FT4 is correlated with risk of MetS and its components [15]. Another study reported that the risk of MetS mostly increased at TSH values below the median and the odds ratio for MetS in the highest FT4 quintile was 1.49 (1.16 1.90) comparing to the lowest FT4 quintile, concluding that TSH and FT4 within normal range were associated with MetS [16].

However, most of these studies were cross-sectional studies and can not discern the cause and effect relationships of THs and MetS. Therefore, we performed a retrospective cohort study to analyze the associations between THs within the normal reference ranges and MetS in a Chinese euthyroid population. We used the cutoff value to divide the cohort into the higher-THs and lower-THs groups, and compared the risk of MetS between the two groups.

## Methods

### Study population

This study was a retrospective cohort study conducted in Shaanxi Province, China. Data were collected from a five-years health examination carried out annually in Xi’an Electric Power Central Hospital from April 2011 to December 2015. (More details about the health examination survey could be seen in Supplementary Material. 1) All Participants agreed to participate and signed informed consent for their data to be analysed with a fully comprehensive understanding of the examination purpose and measurements. Health examination included anthropometric measurements, blood assessments and basic information questionnaires (Details will be provided in the following section). This study was approved by the Ethics Committee of Xi’an Electric Power Central Hospital.

**Fig. 1:**
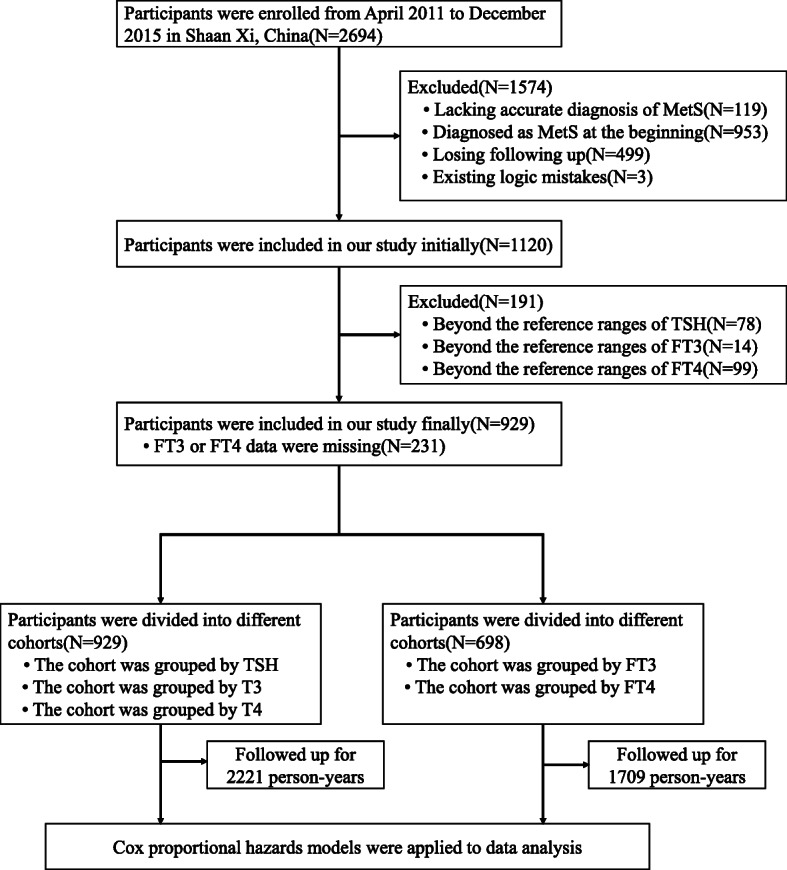
Flowchart of the progress of the retrospective cohort study

The selection process for this cohort is described in Fig. 1. Initially, we collected data from the whole health examined population (2694 individuals) who were not pregnant, had no cancers, no history of thyroid diseases (neither abnormal serum thyroid function state nor abnormal ultrasound results) and no history of taking antithyroid drugs. Participants who had been already diagnosed as MetS at baseline (*n* = 953) or lacked evidence to support an accurate diagnosis of MetS (*n* = 119) were excluded. Then, participants whose follow-ups had not been completed (*n* = 499) or whose data had logical errors (*n* = 3) were excluded. Thus, 1120 participants were selected for this study. Then, participants who had serum levels outside the reference ranges of TSH (< 0.27 mIU/L or > 4.2 mIU/L, *n* = 78), FT3 (< 3.1 pmol/L or > 6.8 pmol/L, *n* = 14) or FT4 (< 12.0 pmol/L or > 22.0 pmol/L, *n* = 99) were excluded. Finally, 929 participants were enrolled in our first cohort. Because the length of follow-up years varied differently among these 929 participants, we calculated the total results. They were followed up for 2221 person-years in total. In these 929 participants, some did not choose to do serum FT3 or FT4 values tests in annually examination(*n* = 231); thus, the first cohort (929 participants followed up by 2221 person-years totally) was investigated only for the relationship between TSH, T3, T4 and MetS.

### Calculating cutoff values for each THs and dividing two cohorts into the higher-TH and lower-TH groups for analysis

To obtain a clear cutoff value for each TH, we performed two steps. Taking TSH as an example: First, in one participant, we calculated the geometric mean of TSH serum values acquired in all his/her annual blood assessments and designated it as his/her TSH representative value. Then, we collected all participant representative TSH values in the cohort and selected the median value as the cutoff value. In addition to TSH, other TH variables were processed in the same way. Eventually, the cutoff values for THs were obtained: TSH, 2.0 mIU/L; T3, 1.9 nmol/L; T4, 117.0 nmol/L; FT3, 4.3 pmol/L; FT4, 16.0 pmol/L. According to the cutoff values, the two cohorts were divided into the higher-TH and lower-TH groups for comparing the incidence of MetS.

### Definitions

Euthyroidism was defined as TSH, FT3 and FT4 levels within the reference ranges. The reference ranges of TSH, FT3 and FT4 were 0.27–4.2 mIU/L, 3.1–6.8 pmol/L and 12.0–22.0 pmol/L, respectively. MetS was defined in accordance with the criteria of the American Heart Association/National Heart, Lung, and Blood Institute (AHA/NHLBI) scientific statements of 2009. People were considered to have MetS when they presented with any three or more of the following five conditions: 1) elevated waist circumference (WC) (Asian criteria (from the World Health Organization) for men > = 90 cm, for women > = 80 cm); 2) elevated TG (> = 1.7 mmol/L) or drug treatment for elevated TG; 3) reduced HDL-C (for men < 1.0 mmol/L, for women < 1.3 mmol/L) or drug treatment for reduced HDL-C; 4) elevated BP (systolic > = 130 mmHg and/or diastolic > = 85 mmHg) or antihypertensive drug treatment in a patient with a history of hypertension; and 5) elevated fasting glucose (> = 100 mg/dL) or drug treatment for elevated glucose.

### Clinical and laboratory measurements

Fasting blood samples were collected from all participants between 8 am and 9 am. Thyroid hormones, including TSH, FT3, FT4, T3 and T4, were all measured by chemiluminescence immunoassay using a Snibe Diagnostic (Maglumi 2000 plus) instrument. WC was measured at the umbilical level when participants stood and breathed normally. BP was measured twice with participants sitting quietly. The 75-g oral glucose tolerance test (OGTT) was conducted in all participants, excluding people who had already been diagnosed with diabetes before or were taking glucose-controlling drugs. Fasting glucose and 2-h glucose (defined as postprandial blood glucose, PBG) were measured by the hexokinase method using a HITACHI 7180 machine and standard reagents. Glycated hemoglobin (GHB) was measured by immunoturbidimetry. Serum TG and total cholesterol (TC) were measured by enzymatic methods. Low-density lipoprotein cholesterol (LDL-C) was measured using the polyvinyl sulfuric acid precipitation method, while HDL-C was measured depending on the chemical precipitation method using standard reagents on the HITACHI 7180 machine from Zybio Company. All biochemical tests were conducted on the day of blood sample drawing. Basic indicators such as age and sex were obtained from written questionnaires. Height and weight were measured through standard methods. BMI (body mass index) was calculated as weight/height^2^ (kg/m^2^).

### Statistical analysis

The description of baseline characteristics depended on the distribution of the parameters assessed by a normality test (Kolmogorov-Smirnov, Shapiro-Wilk) unless generally recognized as normality. The normal distribution parameters, the skewed distribution parameters, and the binary variables were presented as the means (standard deviation, SD), median (interquartile range, IQR), and percentages, respectively. The comparisons between groups were tested by t-test (means±SD), Mann-Whitney U test (median, IQR) and Pearson chi-square test (percentages). All MetS-related variables were updated annually according to health examination results. To reveal the persistent effects of thyroid hormones on the risk of MetS throughout the whole follow-up period, we calculated the geometric mean of THs serum values acquired in all annual blood assessments in every participant. Cox proportional hazards models were used for analysis of the correlations between different thyroid hormone concentrations and the risk of MetS. Confounding factors, including sex, age, BMI, PBG, LDL-C and GHB, were adjusted for. All statistical analyses were performed with SPSS 22.0 (SPSS, Chicago, IL, USA).

## Results

### Basic characteristics of the two cohorts

The clinical and laboratory characteristics of the two cohorts subdivided into the higher-TH and lower-TH groups according to the THs cutoff values are presented in Table 1. The sex composition was presented as the men/women ratio. The age, BMI, WC, TG, HDL-C, DBP, SBP, GLU, PBG, LDL-C, and GHB were presented as the median (interquartile range) values. No significant differences between the higher-TH and the lower-TH groups were found for sex, BMI, HDL-C, DBP, SBP, PBG and GHB. The higher-TSH group showed to be significant older and have a higher TG levels than the lower-TSH group. The higher-T3 group reported to be significantly younger and have a higher TG levels compared with the lower-T3 group. The higher-T4 group showed a significant higher LDL level than the lower-T4 group. WC and GLU level were found significantly higher in the higher-FT3 group than the lower-FT3 group. And participants in the higher-FT4 group were significantly younger than participants in the lower-FT4 group.

**Table 1:** Baseline characteristics of enrolled participants and the association between two groups divided by thyroid function index

Variables^**a**^	Geometric Mean of Thyroid Function Index During Follow-up years									
	Basic cohort (929 participants/2221 person-years)						Second cohort (698 participants/1709 person-years)			
	TSH (mIU/L)		T3 (nmol/L)		T4 (nmol/L)		FT3 (pmol/L)		FT4 (pmol/L)	
	< 2.0	> 2.0	< 1.9	> 1.9	< 117	> 117	< 4.3	> 4.3	< 16	> 16
Number of participants	477	452	444	485	453	476	339	359	313	385
SEX^a3^ (Man/women)	384/93	319/133	320/124	383/102	338/115	365/111	243/96	306/53	235/78	314/71
Age^a2^ (years)	47.8 (13.9)	52.5 (14.3) ^b^	51.3 (14.6)	49.0 (13.9) ^b^	49.3 (14.1)	50.9 (14.4)	44.8 (8.5)	43.9 (9.9)	45.2 (8.7)	43.7 (9.7) ^b^
BMI (kg/m^2)^	23.05 (3.31)	23.05 (3.68)	22.91 (3.63)	23.25 (3.47)	23.03 (3.26)	23.14 (3.77)	23.23 (3.86)	23.41 (3.32)	23.52 (3.58)	23.20 (3.42)
WC (cm)	85 (10)	85 (11)	85 (12)	85 (11)	85 (11)	85 (11)	85 (11)	86 (9) ^b^	86 (9)	86 (10)
TG (mmol/l)	1.2 (0.7)	1.3 (0.7) ^b^	1.2 (0.7)	1.3 (0.7) ^b^	1.2 (0.7)	1.3 (0.7)	1.3 (0.7)	1.3 (0.8)	1.4 (0.7)	1.3 (0.7)
HDL-C (mmol/l)	1.3 (0.4)	1.3 (0.4)	1.3 (0.4)	1.3 (0.4)	1.3 (0.3)	1.3 (0.4)	1.3 (0.4)	1.3 (0.4)	1.3 (0.3)	1.3 (0.4)
DBP (mmHg)	74 (11)	75 (15)	76 (14)	74 (13)	74 (14)	75 (13)	74 (13)	75 (13)	75 (14)	74 (13)
SBP (mmHg)	117 (18)	118 (19)	117 (21)	118 (17)	116 (19)	118 (18)	114 (16)	116 (15)	115 (15)	116 (16)
GLU (mmol/l)	5.3 (0.6)	5.3 (0.5)	5.3 (0.6)	5.3 (0.6)	5.3 (0.6)	5.3 (0.6)	5.2 (0.6)	5.3 (0.6) ^b^	5.3 (0.6)	5.3 (0.5)
PBG (mmol/l)	6.5 (1.7)	6.7 (1.8)	6.5 (1.9)	6.7 (1.7)	6.6 (1.8)	6.6 (1.9)	6.3 (1.6)	6.4 (1.7)	6.3 (1.6)	6.4 (1.8)
LDL (mmol/l)	2.5 (1.0)	2.5 (0.8)	2.5 (0.9)	2.5 (0.9)	2.4 (0.9)	2.6 (0.9) ^b^	2.4 (0.8)	2.5 (0.9)	2.5 (0.9)	2.4 (0.8)
GHB (%)	4.4 (0.7)	4.4 (0.6)	4.4 (0.6)	4.4 (0.7)	4.4 (0.7)	4.4 (0.7)	4.4 (0.6)	4.4 (0.5)	4.4 (0.5)	4.4 (0.6)

*Abbreviations*: *BMI* body mass index, *BP* blood pressure, *DBP* diastolic blood pressure, *GLU* glucose, *GHB* glycated hemoglobin, *HDL* high density lipoprotein, *LDL* low density lipoprotein, *PBG* postprandial blood glucose, *SBP* systolic blood pressure, *TG* triglyceride, *WC* waist circumference

^a^ Values are median (interquartile range) or means (standard deviations) ^a2^ or ratio (men/women)^a3^ to the age distribution of the study population

^b^ Indicates that baseline characteristics divided by present thyroid function index were significantly different from each other (*p* < 0.05, Chi-square test, T-test, Non-parametric test)

### The associations between THs and the incidence of MetS

The associations between TSH, T3, T4, FT3, and FT4 and the incidence of MetS are indicated in Table 2. The cutoff values for THs were obtained: TSH, 2.0 mIU/L; T3, 1.9 nmol/L; T4, 117.0 nmol/L; FT3, 4.3 pmol/L; FT4, 16.0 pmol/L. In first cohort (929 participants/2221 person-years), the number of participants who presented with MetS/ their total follow-up years ratio, in the higher-TSH, lower-TSH, higher-T3, lower-T3, higher-T4 and lower-T4 groups were 141/1057, 113/1164, 133/1056, 121/1165, 132/1156 and 122/1065, respectively. In the second cohort (698 participants/1709 person-years), the ratio in the higher-FT3, lower-FT3, higher-FT4 and lower-FT4 groups were 95/870, 85/839, 80/956, and 100/753, respectively.

**Table 2:** Comparison of the incidence of metabolic syndrome between the higher-TH and the lower-TH groups

	Dividing cohorts into the higher-TH and the lower-TH groups according to cutoff values		*P*-value
TSH (mIU/L)	≤2.0	> 2.0	
Participants/person-years	113/1164	141/1057	
Model 1^a^	1.00	1.358 (1.060, 1.740)	0.015
Model 2^b^	1.00	1.308 (1.016, 1.683)	0.037
Model 3^c^	1.00	1.263 (0.972, 1.640)	0.081
T3 (nmol/L)	≤1.9	> 1.9	
Participants/person-years	121/1165	133/1056	
Model 1^a^	1.00	1.000 (0.781, 1.279)	0.997
Model 2^b^	1.00	1.016 (0.793, 1.302)	0.899
Model 3^c^	1.00	1.005 (0.781, 1.294)	0.969
T4 (nmol/L)	≤117	> 117	
Participants/person-years	122/1065	132/1156	
Model 1^a^	1.00	1.004 (0.785, 1.284)	0.975
Model 2^b^	1.00	0.967 (0.756, 1.238)	0.792
Model 3^c^	1.00	1.003 (0.779, 1.290)	0.984
FT3 (pmol/L)	≤4.3	> 4.3	
Participants/person-years	85/839	95/870	
Model 1^a^	1.00	1.078 (0.804, 1.445)	0.615
Model 2^b^	1.00	1.012 (0.753, 1.361)	0.937
Model 3^c^	1.00	1.054 (0.778, 1.429)	0.734
FT4 (pmol/L)	≤16	> 16	
Participants/person-years	100/753	80/956	
Model 1^a^	1.00	0.636 (0.474, 0.854)	0.003
Model 2^b^	1.00	0.619 (0.460, 0.832)	0.001
Model 3^c^	1.00	0.622 (0.458, 0.846)	0.002

^a^ Estimates were calculated in Cox proportional hazards models. Model 1, unadjusted

^b^ Model 2 was adjusted for sex and age based on model 1

^c^ Model 3 was further adjusted for BMI, PBG, LDL-C and GHB based on model 2

Cox proportional hazards models were used to analyze the incidence of MetS between the higher-TH and the lower-TH groups: model 1, unadjusted; model 2, adjusted for sex and age; model 3, adjusted for sex, age, BMI, PBG, LDL and GHB. The incidence of MetS was reflected by the hazard ratio (HR).

Adjusted for sex, age, BMI, PBG, LDL and GHB in Model 3, the hazard ratios (95% confidence intervals) that predicts the incidence of MetS in the higher-TH group compared with the lower-TH group among the TSH, T3, T4, FT3 and FT4 were 1.263 (0.972–1.640; *P* = 0.081); 1.005 (0.781–1.294; *P* = 0.969); 1.003 (0.779–1.290, *P* = 0.984); 1.054 (0.778–1.429, *P* = 0.734); and 0.622 (0.458–0.846, *P* = 0.002), respectively. Obviously, only FT4 was significantly associated with MetS and low normal FT4 was an independent risk factor for MetS in all three Models. However, when unadjusted in Model 1 and adjusted for sex and age in Model 2, the participants in higher-TSH group both showed significantly higher incidence of MetS (Model 1: 1.358(1.060, 1.740) *P* = 0.015; Model 2: 1.308(1.016, 1.683) *P* = 0.037). And there were no significant correlations reflected in the HRs of T3, T4 and FT3 in any of the three models.

### The incidence of MetS between different FT4/TSH groups

As shown in Table 3, we used the combination of cutoff values of FT4 and TSH to divide the first cohort into four groups: lower-TSH/higher-FT4 group, lower-TSH/lower-FT4 group, higher-TSH/higher-FT4 group and higher-TSH/lower-FT4 group. The incidence of MetS in these four groups were analyzed by Cox proportional hazards models and adjusted in three models in the same way as mentioned above. The incidence of MetS in the lower-normal thyroid function status (higher-TSH/lower-FT4 group) is significantly the highest. (Model 1: 2.066(1.375, 3.106) *P* = 0.005; Model 2: 2.154(1.428, 3.251) *P* = 0.003; Mode 3:2.131(1.380,3.291) *P* = 0.006). And, the risk of MetS in the lower-TSH/higher-FT4 group (Mode 3: 1.874(1.199, 2.928) *P* = 0.006) is significantly higher than the risk of MetS in the higher-TSH/lower-FT4 group (Mode 3: 1.600(1.010, 2.536) *P* = 0.006).

**Table 3:** Comparison of the incidence of metabolic syndrome in different TSH and FT4 groups

	Geometric Mean of Thyroid Function Index During Observation Period, mIU/L				*P*-value
	TSH (≤2.0)		TSH (> 2.0)		
	FT4(> 16)	FT4(≤16)	FT4(> 16)	FT4(≤16)	
Cases/person-years	40/575	45/378	40/381	55/375	
Model 1^a^	1.00	1.706 (1.113, 2.615)	1.502 (0.969, 2.329)	2.066 (1.375, 3.106)	0.005
Model 2^b^	1.00	1.755 (1.145, 2.691)	1.522 (0.982, 2.360)	2.154 (1.428, 3.251)	0.003
Model 3^c^	1.00	1.874 (1.199, 2.928)	1.600 (1.010, 2.536)	2.131 (1.380,3.291)	0.006

^a^ Estimates are calculated in Cox proportional hazards models. Model 1, unadjusted

^b^ Model 2 was further adjusted for sex and age based on model 1

^c^ Model 3 was further adjusted for BMI, PBG, LDL and GHB based on model 2

### Survival analysis of the incidence of MetS in FT4 groups, TSH groups and FT4/TSH groups

**Fig. 2:**
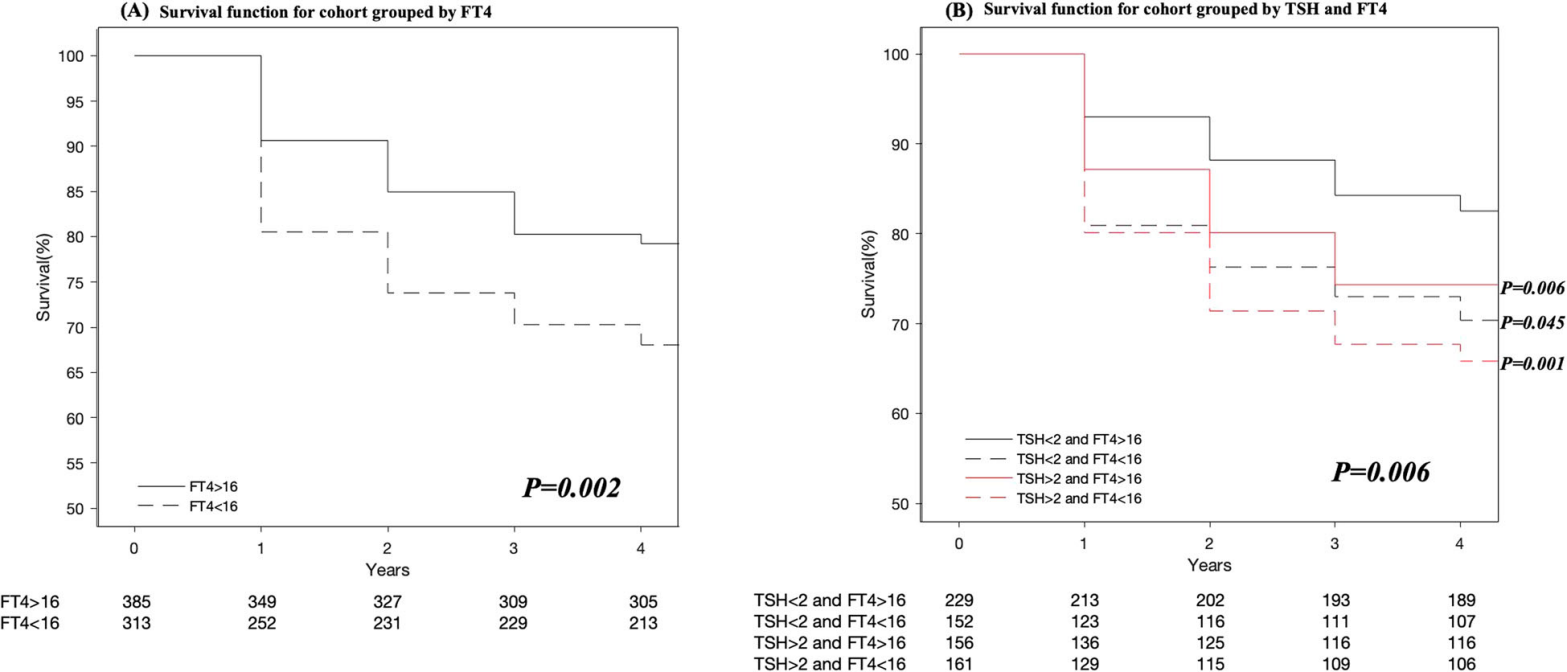
Survival function for cohort groups. **a** Survival function for cohort grouped by FT4. **b** Survival function for cohort grouped by TSH and FT4

The survival analysis curves intuitively showed the change of the ratio of participants without MetS to the total population in different FT4 groups, TSH groups (Fig. 2a), and FT4/TSH groups (Fig. 2b**)** during their respective follow-up years. All population were healthy without MetS at the beginning. At the end of the follow-up, the number of subjects without MetS were 305, 213, 189, 107, 116 and 106 in the higher-FT4, lower-FT4, lower-TSH/higher-FT4, lower-TSH/lower-FT4, higher-TSH/higher-FT4 and higher-TSH/lower-FT4 groups, respectively.

## Discussion

In this retrospective cohort study, we acquired cutoff values to assess the associations between thyroid hormones (TSH, T3, T4, FT3 and FT4) and the incidence of MetS in a euthyroid population. We observed that after adjustment for sex, age, BMI, PBG, LDL and GHB, lower normal FT4 (FT4 ≤ 16.0 pmol/L), is an independent risk factor for MetS, and participants in lower-normal thyroid status (FT4 ≤ 16.0 pmol/L and TSH > 2.0 mIU/L) have a higher risk of developing MetS. Our results indicate that the combination use of TSH and FT4 could be more dependable compared with the assessments based on only FT4 or TSH for the prediction of the risk of MetS.

In fact, among the variety of studies analyzing the relationship between thyroid hormones and the MetS, FT4 and TSH are the two most studied hormones. Lower normal FT4 was reported to be associated with an increased risk of MetS by Lin et al. [17], and to be significantly correlated with lipids and higher insulin resistance and finally related to four of the five MetS components by Roos et al. [18]. However, although these two studies could reveal the association between lower FT4 within normal reference range and MetS, they were both cross-sectional studies, unable to discern the cause and effect relationships of FT4 and MetS. Besides, to explore the differences in the incidence of MetS caused by slight change in FT4 levels, Lin et al. [17] decided to divide circulating FT4 concentrations into quartiles, while Roos et al. [18] chose tertiles and we used dichotomy method. Interestingly, our FT4 cutoff value (16.0 pmol/L) is almost the median of the 95% reference range of FT4 (11.70 to 20.28 pmol/L) in one study based on clinical big data including 20,077 Chinese individuals [19]. This may be a coincidence and need further exploration, but it indicated that the FT4 levels in the present study were normally distributed and had no significant difference with other big data.

As for TSH, higher normal TSH has been widely reported to be associated with MetS. A Germany study found that participants with a TSH in the upper normal range (2.5–4.5mIU/L) had a 1.7-fold increased risk for MetS than those with a TSH in the lower normal range (0.3–2.5mIU/L) [20], while another study demonstrated that after adjusting for age and gender, people over 40 years old with higher normal TSH levels (2.5–5.0mIU/L) had a 1.2-fold increased risk of MetS than those with lower normal TSH levels (0.35–2.5mIU/L) [14]. We acquired the cutoff values for TSH as 2.0 mIU/L, a little bit lower than the upper normal recommended value (2.5 mIU/L) from the National Academy of Clinical Biochemistry (NACB) criteria [21], but in line with an Italian study using 2.0 mIU/L as the cut-off point for TSH levels [22] and close to another study reporting TSH ranged from 0.3 to 4.9 mIU/L with a median of 2.1 mIU/L [23]. The NACB recommended value was acquired on the foundation that 95% of normal Caucasian subjects had TSH levels < 2.5 mIU/L, thus it suggests to downregulate the upper normal limit of TSH from 4.5 mIU/L to 2.5 mIU/L. Since ethnic differences may occur between our Chinese participants and the Caucasian population, the deviation could be understood and the identification of a suitable and convenient TSH cutoff value still worth further discussion. Our results are partly consistent with the above researches [14, 20], in the present study, participants in the higher-TSH group (TSH > 2mIU/L) showed significantly higher incidence of MetS when unjusted in Model 1 and adjusted for sex and age in Model 2, however, after adjusting for sex, age, BMI, PBG, LDL and GHB in Model 3, higher TSH showed no significant correlation with MetS. This may be attributed to the interaction effect between TSH and BMI, PBG, LDL and GHB.

As for lower-normal thyroid function status composed of both lower-normal FT4 and higher-normal TSH, which is exactly our condition. We found that among the four TSH/FT4 groups, the risk of MetS in the higher-TSH/lower-FT4 (FT4 ≤ 16.0 pmol/L and TSH > 2.0 mIU/L) is the highest, compared to the lower-TSH/higher-FT4 group. And among the remaining three groups, the risk of MetS in the lower-TSH/lower-FT4 group was the second highest, higher than that in the higher-TSH/higher-FT4 group. From this result, it seems that lower-FT4 has a greater effect on MetS than higher-TSH. This conclusion agreed to a cohort study which indicated that although TSH has been recognized as a highly sensitive measure of thyroid function, however, FT4 could be more reliable in reflecting thyroid status, because circulating FT4 affects peripheral thyroid activity [24]. Thus we propose that the combined use of TSH and FT4 is a more advanced and dependable approach compared with the assessment based on only FT4 or TSH, because it can reflect the thyroid function more accurately.

The mechanism how lower normal thyroid status may have adverse effect on metabolic indicators and increase the risk of MetS even in euthyroid population had not been clearly explained, however, there exist some reasonable explanations: First, insulin resistance is assumed to be both the key feature, and the major underlying process for MetS, and lower normal thyroid function, especially lower normal FT4 has been demonstrated to be associated with insulin resistance [17, 25]; Second, high WC presenting abdominal obesity is one of the five components of MetS. THs control the intake and expenditure of energy, while lower normal thyroid function may lead to a decrease in energy consumption and accumulation of adipose tissue, which attributes to abdominal obesity; Thirdly, THs also regulate lipid metabolism through directly acting on liver and adipose tissue and the hypothalamic control of lipid metabolism [26, 27]. Lower normal thyroid function may confer increased plasma PCSK9 levels, regulate LDL receptor expression and contribute to affect cellular cholesterol trafficking [28]. And lower normal thyroid function may also negatively affect the ability of HDL which protect LDL from oxidative modification and impair the HDL anti-oxidative capacity [28]; Fourthly, THs also play an important role in maintaining cardiovascular homeostasis. Subtle changes in thyroid hormone concentrations, like subclinical hypothyroidism or hyperthyroidism, could lead to a significant increase in vascular morbidity and mortality [29, 30], while lower normal thyroid function, especially lower FT4 levels may increase systemic vascular resistance, decrease arterial compliance and affect BP; Finally, lower normal thyroid function is reported to be a risk factor for diabetes, especially in individuals with prediabetes [31], the mechanism of which could be attributed to a decreased insulin sensitivity and glucose tolerance.

Our study confirmed that lower normal thyroid function is associated with a higher risk of MetS, however, we mainly focused on the diagnosis of the complete presentation of MetS, but ignored the associations between THs and the five components of MetS. Lack of analysis on the incidence of each components of MetS between the higher-TH and the lower-TH groups is a main shortcoming of our study. Another limitation is that we did not measure thyroid antibodies, which might be a potential influencing factor for MetS. For example, Michalopoulou et al. [32] found that subjects with high-normal TSH (2.0 to 4.0 mIU/L) combined with positive thyroid autoantibodies may have subclinical hypothyroidism manifesting with elevated cholesterol levels, and Siemińska L et al. [33] suggested that subjects with upper quartile of TSH had significantly higher thyroid autoantibody levels than subjects with lower quartile. Further researches with deeper analysis will be needed from our team in the future.

## Conclusion

In summary, we show that lower-normal FT4 (FT4 ≤ 16.0 pmol/L) is an independent risk factor for MetS, and through a combination of TSH and FT4, instead of only one thyroid hormone indicator, we found that lower-normal thyroid function (TSH > 2 mIU/L and FT4 ≤ 16 pmol/L) is associated with a higher risk of MetS. (2.131vs 1.0 (1.380,3.291), *P* = 0.006). More larger sample prospective studies will be needed to assess a possible role for intervention in the slight migration of thyroid function at an earlier stage for preventing MetS in the future.

## Supplementary Information

**Additional file 1: Supplementary Material 1.** Health examination survey.

## Data Availability

The datasets used and/or analyzed in this study are available from the corresponding author on reasonable request.

## Abbreviations

THs: Thyroid hormones
MetS: Metabolic syndrome
T3: Triiodothyronine
T4: Thyroxine
FT3: Free triiodothyronine
FT4: Free thyroxine
AHA/NHLBI: American Heart Association/National Heart, Lung, and Blood Institute
CVD: Cardiovascular diseases
BP: High blood pressure
TG: Triglyceride
HDL-C: High-density lipoprotein cholesterol
BMI: Body mass index
SCH: Subclinical hypothyroidism
WC: Waist circumference
OGTT: Oral glucose tolerance test
PBG: Postprandial blood glucose
GHB: Glycated hemoglobin
SD: standard deviatio
IQR: Interquartile range
NACB: National Academy of Clinical Biochemistry
