# Supplementary material for: Lower normal free thyroxine is associated with a higher risk of metabolic syndrome: a retrospective cohort on Chinese population
Source: BMC Endocr Disord. 2021 Mar 4;21:39. doi: 10.1186/s12902-021-00703-y (PMC7934401; doi:10.1186/s12902-021-00703-y)
Supplement: Supplementary file 1 — Additional file 1: Supplementary Material 1. Health examination survey. [file 12902_2021_703_MOESM1_ESM.docx]

**Health Examination Survey**

Health examination institution: Xi'an Electric Power Central Hospital

Health examination date :___

**Health Examination items:**

1) Health Questionnaire

2) Physical examination

3) Special Examination

| Basic information | Name | |  | | | | Gender | ○ men ○ women | | | | | Date of birth | |  | |
| --- | --- | --- | --- | --- | --- | --- | --- | --- | --- | --- | --- | --- | --- | --- | --- | --- |
|  | Current area | |  | | | | | □ agree to use personal cyberspace  □ consent to health intervention | | | | | | | | |
|  | Phone number | |  | | | | | E-mail | |  | | | | | | |
|  | ABO blood type | | ○ Type A ○ Type B  ○ Type AB ○ Type O | | | | | Marriage | | ○ unmarried ○ married ○ widowed ○ divorced | | | | | | |
|  | Type of occupation | | ○ Administration ○ Professional Technical ○ Service ○ Commercial Services ○ Agriculture, Forestry, Animal Husbandry and Fisheries ○ Workers ○ Soldiers, Retired and others | | | | | | | | | | | | | |
| Illness | Relationship \| disease | | Hypertension\|Diabetes \| Coronary heart disease\|Hyperlipidemia \| Obesity\|Stroke\|Lung cancer\|Prostate cancer\|Breast cancer\|Osteoporosis \|Liver cancer\|Alzheimer \|Gastric cancer\|Chronic obstructive pulmonary disease | | | | | | | | | | | | | |
|  | Myself | | □ □ □ □ □ □ □ □ □ □ □ □ □ □ | | | | | | | | | | | | | |
|  | Fathers | | □ □ □ □ □ □ □ □ □ □ □ □ □ □ | | | | | | | | | | | | | |
|  | Mother | | □ □ □ □ □ □ □ □ □ □ □ □ □ □ | | | | | | | | | | | | | |
|  | Grandparents | | □ □ □ □ □ □ □ □ □ □ □ □ □ □ | | | | | | | | | | | | | |
|  | Grandparents | | □ □ □ □ □ □ □ □ □ □ □ □ □ □ | | | | | | | | | | | | | |
| 1. Health Questionnaire: | | | | | | | | | | | | | | | | |
| 1. Physical examination: | | | | | | | | | | | | | | | | |
| Basic projects | Temperature | Pulse | | Heart rate | | Blood pressure | | | Height | | Weight | | | BMI | | Waist circumference |
| Required | Fasting blood glucose (mmol/L) | | | | | | | | Low density lipoprotein cholesterol (mmol/L) | | | | | | | |
|  | White blood cell count (109L //） | | | | | | | | High density lipoprotein cholesterol (mmol/L) | | | | | | | |
|  | Red blood cell count (109L //） | | | | | | | | Alanine aminotransferase (U/L) | | | | | | | |
|  | Hemoglobin (g/L) | | | | | | | | Total bilirubin (μmol/L) | | | | | | | |
|  | Platelet count (109/L） | | | | | | | | Blood urea nitrogen (mmol/L) | | | | | | | |
|  | Total cholesterol (mmol/L) | | | | | | | | Blood creatinine (μmol/L) | | | | | | | |
|  | Triacylglycerol (mmol/L) | | | | | | | | Blood uric acid (μmol/L) | | | | | | | |
| Auxiliary examination | ECG | | | | | | | | | | | | | | | |
|  | Chest ultrasound | | | | | | | | | | | | | | | |
|  | X chest radiographs | | | | | | | | | | | | | | | |
|  | **Thyroid ultrasound** | | | | | | | | | | | | | | | |
| Optional projects | Blood routine test | | | | Thyroid hormone test (including three hormones) | | | | | | | Electrolytes test | | | | |
|  | Urine test | | | | Thyroid hormone test (including five hormones) | | | | | | | Infectious indicators test | | | | |
|  | Fecal routine test | | | | Hepatitis series (A, B, C, E) test | | | | | | | Glycosylated hemoglobin | | | | |
|  | Blood cell quintiles | | | | Connective tissue test | | | | | | | β human chorionic gonadotropin | | | | |
|  | Bone Metabolism test | | | | Autoantibody Spectrometry | | | | | | | OGTT | | | | |
|  | Kidney function test | | | | Sex hormone set | | | | | | | Biood Coagulation test | | | | |
|  | Blood gas analysis | | | | Ten tumor markers (female) | | | | | | | Ten tumor markers (male) | | | | |
| 3) Special Examination | | | | | | | | | | | | | | | | |

**Health Questionnaire**

Health examination institution: Xi'an Electric Power Central Hospital

Name, sex, age，marital status

ID number unit

**Part 1.** Family history :(if your immediate family has the following diseases, please type √ in 口)

1. High blood pressure 口 father口mother口 grandparents口brothers and sisters 口

Diagnosis time_____treatment effect: cured口 on the mend 口not cured口

1. Coronary heart disease口 father口mother口 grandparents口brothers and sisters 口

Diagnosis time_____treatment effect: cured口 on the mend 口not cured口

1. Diabetes 口 father口mother口 grandparents口brothers and sisters 口

Diagnosis time_____treatment effect: cured口 on the mend 口not cured口

1. Cerebrovascular disease 口 father口mother口 grandparents口brothers and sisters 口

Diagnosis time_____treatment effect: cured口 on the mend 口not cured口

1. Thyroid disease 口 father口mother口 grandparents口brothers and sisters 口

Diagnosis time_____treatment effect: cured口 on the mend 口not cured口

1. Hyperlipidemia 口 father口mother口 grandparents口brothers and sisters 口

Diagnosis time_____treatment effect: cured口 on the mend 口not cured口

1. Cancer 口 father口mother口 grandparents口brothers and sisters 口

Diagnosis time_____treatment effect: cured口 on the mend 口not cured口

**Part 2.** Past medical history: what diseases have I suffered in the past?

1. Disease name_____ ,diagnosis time_____treatment effect: cured口 on the mend 口not cured口

2. Disease name_____ ,diagnosis time_____treatment effect: cured口 on the mend 口not cured口

3. Disease name_____ ,diagnosis time_____treatment effect: cured口 on the mend 口not cured口

**Part 3.** Menstrual history (Female only): menstruation: menarche years, cycle days, each menstrual cycle days.

History of marriage and childbearing :(Female only)

Fertility: term delivery, premature delivery, spontaneous abortion, artificial abortion.

Children: Yes 口No 口 Sex ___Age___ School or not 口

**Part 4.**

History of iodized salt application: Yes 口No 口 start time___, estimated daily dosage___g/day

Smoking history: Yes 口No 口 start smoking time: ___, smoking cessation time: ___, smoking volume: ___branch / day

Drinking history: Yes 口No 口 start drinking time___year, ___month,

Alcohol consumption: ___g/day (liquor)

History of thyroid disease: Have you ever taken amiodarone tablets in the past 3 months? Yes 口No 口

Have you had thyroid surgery? Yes 口No 口

Have you ever taken or are you taking thyroxine tablets ? Yes 口No 口

Have you ever taken or are taking tabazole tablets or propylthiouracil tablets? Yes 口No 口

**Part 5.**

History of hypertension and treatment

Have you ever taken or are taking antihypertensive drugs? Yes 口No 口; Name of specific drug

Have you ever taken blood pressure drops in the last month? Yes 口No 口

History of coronary heart disease and treatment

Have you ever taken or are taking antihypertensive drugs? Yes 口No 口;Name of specific drug

Have you ever taken coronary heart disease medicine in the last month? Yes 口No 口

History of Hyperlipidemia and Treatment

Have you ever taken or are taking antihypertensive drugs? Yes 口No 口;Name of specific drug

Have you ever taken hypolipidemic drugs in the last month? Yes 口No 口

History of Diabetes and Treatment

Have you ever taken or are taking antihypertensive drugs? Yes 口No 口;Name of specific drug

Have you ever taken hypoglycemic drugs in the last month? Yes 口No 口

Gout and history of treatment

Do you have a history of pain in the big toe joint or have you been diagnosed with a history of gout?Yes 口No 口

Have you ever taken or are you taking any glitzol or benzbromarone tablets? Yes 口No 口

Have you ever taken painkillers in the last three years?Yes 口No 口

Career history: job type_____

Exposure to hazardous factors: ____hours of exposure

**Thyroid ultrasound:**

Thyroid ultrasound was performed by two trained professional ultrasound technicians. The diagnostic criteria for normal thyroid ultrasound include the following:

1. The echo of the whole gland is uniform and the light spots are fine and dense;
2. No local nodules;
3. No thyroid enlargement can meet the following criteria: left and right diameter (transverse diameter)<2 cm, thyroid anteroposterior diameter should be 2 cm, thyroid isthmus anteroposterior diameter <0.5 cm. The goiter is defined as the product of the length, width and thickness of the thyroid gland, and then multiplied by the coefficient of 0.479 under the calibration J. The calculated volume values are as follows: female >19.4 ml, male >25.6 ml, can be goiter;
4. There was no obvious decrease and absence of thyroid gland, no ectopic thyroid gland;
5. No adenoma;
6. No local cyst, no calcification and halo.
